# Dynamic changes of the proportion of HLA-DR and CD38 coexpression subsets on T lymphocytes during IFN-based chronic hepatitis B treatment

**DOI:** 10.3389/fimmu.2022.1116160

**Published:** 2023-01-24

**Authors:** Yanjie Lin, Ge Shen, Si Xie, Xiaoyue Bi, Huihui Lu, Liu Yang, Tingting Jiang, Wen Deng, Shiyu Wang, Lu Zhang, Yao Lu, Yuanjiao Gao, Hongxiao Hao, Shuling Wu, Ruyu Liu, Min Chang, Mengjiao Xu, Leiping Hu, Xiaoxue Chen, Ronghai Huang, Minghui Li, Yao Xie

**Affiliations:** ^1^ Department of Hepatology Division 2, Peking University Ditan Teaching Hospital, Beijing, China; ^2^ Department of Hepatology Division 2, Beijing Ditan Hospital, Capital Medical University, Beijing, China; ^3^ Division of Hepatology, Hepato-Pancreato-Biliary Center, Beijing Tsinghua Changgung Hospital, School of Clinical Medicine, Tsinghua University, Beijing, China; ^4^ Department of Obstetrics and Gynecology, Wuhan Children’s Hospital (Wuhan Maternal and Child Healthcare Hospital), Tongji Medical College, Huazhong University of Science and Technology, Wuhan, China; ^5^ Department of General Surgery, Beijing Ditan Hospital, Capital Medical University, Beijing, China

**Keywords:** chronic hepatitis B, interferon, HLA-DR, CD38, plateau phase, intermittent therapy

## Abstract

**Background:**

To investigate the changes of human leukocyte antigen DR (HLA-DR) and CD38 coexpression subsets on T lymphocytes following interferon (IFN) therapy for those who have chronic hepatitis B (CHB).

**Methods:**

A prospective cohort of CHB patients participated in this study. CHB patients without IFN treatment (including naïve and nucleoside [nucleotide] analogs [NAs]-treated patients) were given pegylated interferon alfa (Peg-IFNα) treatment. Peripheral blood samples were taken at baseline, 4 weeks and 12-24 weeks of Peg-IFNα treatment. For the patients who entered the Peg-IFNα plateau phase due to the stagnation of the decrease in HBsAg, and Peg-IFNα was discontinued and Peg-IFNα therapy was resumed after an interval of 12-24 weeks. During the interval, they received first-line NAs treatment. Peripheral blood samples were collected at the baseline of the plateau phase, 12-24 weeks of intermittent treatment, and 12-24 weeks of Peg-IFNα retreatment. The peripheral blood samples were taken to determine virological, serological and biochemical indices of hepatitis B virus (HBV), and T lymphocyte related phenotypes were detected using flow cytometry.

**Results:**

In the process of long-term treatment of Peg-IFNα, the percentage of HLA-DR^+^CD38^dim^ subsets increased significantly at first, then decreased gradually, while the percentage of HLA-DR^+^CD38^hi^ subsets markedly increased. During long-term Peg-IFNα treatment, there was a considerable negative correlation between HBsAg and the HLA-DR^+^CD38^hi^ subset percentage. The persistent high proportion of HLA-DR^+^CD38^hi^ subsets was related to the occurrence of Peg-IFNα plateau phase. After Peg-IFNα intermittent treatment, the percentage of HLA-DR^+^CD38^hi^ subsets decreased significantly. After Peg-IFNα retreatment, the level of HBsAg began to decrease again. At the same time, the percentage of HLA-DR^+^CD38^hi^ subsets significantly increased, but it was still lower than that at the baseline level.

**Conclusions:**

The spectrum of HLA-DR and CD38 coexpression subsets on T lymphocytes changed during the long-term treatment of IFN. The establishment of the IFN plateau phase was linked to the persistence of a considerable proportion of HLA-DR^+^CD38^hi^ subsets on T lymphocytes. IFN intermittent treatment could significantly reduce the proportion of HLA-DR^+^CD38^hi^ subsets, helping regain the antiviral efficacy of IFN during IFN retreatment.

## Introduction

Millions of people, especially those in China, are infected with the chronic hepatitis B virus (HBV). Around 86 million people in China have chronic HBV infection, including 30 million who have chronic hepatitis B (CHB), and 400 thousand people die from HBV-related causes per year (1, 2). In addition, HBV infection accounts for 77% and 84% of patients with liver cirrhosis and liver cancer in China, respectively (3). The most crucial step in slowing the disease’s course and enhancing these patients’ long-term prognosis is antiviral medication (4, 5). Two efficient antiviral medications for the treatment of CHB are interferon (IFN) and nucleoside (nucleotide) analogs (NAs). In comparison to NAs, IFN regulates the immune system in addition to acting as a direct antiviral, which plays a more and more important role in antiviral therapy (6–9).

However, although many studies have indicated that long-term IFN antiviral therapy increases hepatitis B surface antigen (HBsAg) clearance, which helps to achieve long-lasting immune control and clinical cure (10, 11). There are still a considerable number of patients who are unable to clear HBsAg from the long-term treatment of IFN. After the patient’s HBsAg level plateaued during IFN therapy, the continued use of IFN for antiviral therapy could not further reduce the HBsAg level (9). It is only after intermittent treatment with IFN that HBsAg levels begin to decrease again (12).

Natural killer (NK) lymphocytes, dendritic lymphocytes, and T lymphocytes all participate in the immune system’s ability to eliminate HBV, with T lymphocyte activation playing the most significant role in virus clearance (13–15). Immature T lymphocytes express the transmembrane glycoproteins human leukocyte antigen DR (HLA-DR) and CD38, which are reexpressed during the T lymphocyte immunological response. Therefore, HLA-DR and CD38 expression on T lymphocytes’ surface, especially the coexpression of both, can well reflect the state of immune activation (16–18). According to the study, the absolute number of CD8^+^ T lymphocytes declined dramatically while receiving long-term IFN therapy, and they primarily lost subsets that were in the stage of terminal differentiation (19). However, this study did not further explore the dynamic changes of phenotypes and functions related to T lymphocyte activation and depletion during long-term treatment of IFN. In addition, there are few reports on the dynamic changes of T lymphocyte-related phenotypes in IFN intermittent therapy for patients reaching plateau phase.

This study’s major goal is to investigate how HLA-DR and CD38 coexpression subsets proportion on T lymphocytes fluctuates dynamically throughout IFN-based treatment of CHB. The findings of this study will aid in explaining why IFN antiviral therapy’s efficiency varies at different phases.

## Materials and methods

### Study design

CHB Patients hospitalized at Beijing Ditan Hospital’s Second Department of Hepatology between June 2021 and August 2022 made up the prospective cohort. According on their course of therapy, the patients were categorized into three groups: The Naïve group consisted of CHB patients who had never undergone antiviral medication, while patients who had only ever undergone first-line NAs antiviral medication made up the NA-treated group. Patients who had previously undergone pegylated interferon alfa (Peg-IFNα) therapy were enrolled in the Plateau group due to the failure of HBsAg to continuously decrease.

In the Naïve group, patients began to receive subcutaneous injection of Peg-IFNα-2a 180 μg/week, and selected first-line NAs antiviral drugs (entecavir [ETV] 0.5 mg/day or tenofovir disoproxil fumarate [TDF] 300 mg/day or tenofovir alafenamide fumarate [TAF] 25 mg/day) according to their clinical data and personal wishes for combined treatment. Peg-IFNα-2a 180 μg/week was administered subcutaneously to patients in NA-treated group on the basis of NAs antiviral drugs. Blood samples were taken from patients in Naïve group and NA-treated group at baseline, 4 week and 12-24 weeks after IFN treatment. The HBV virological and serological indices, biochemical indices, and phenotypes associated with T lymphocytes were identified. In the Plateau group, when HBsAg decreased to the plateau phase, the treatment of IFN was suspended and entered into intermittent treatment for 12-24 weeks, during which only ETV (0.5 mg/day) or TDF (300 mg/day) or TAF (25 mg/day) was used to maintain the antiviral efficacy, and after the interval, IFN was retreated on the basis of NAs. Blood samples were taken from patients in Plateau group at the baseline of plateau, during intermittent treatment of 12-24 weeks and during 12-24 weeks of IFN retreatment. The HBV virological and serological indices, biochemical indices, and phenotypes associated with T lymphocytes were identified. In addition, liver ultrasonography was performed every 12-24 weeks at baseline and during the follow-up period.

In addition, the comparison between the Plateau group and its corresponding control group (that is, patients who continued to receive IFN treatment when HBsAg droped to a plateau) could more accurately portray the advantages of IFN intermittent therapy and the relationship between the changes of T lymphocyte-related surface markers and clinical efficacy during this period. However, patients who HBsAg levels decreased < 1 lg IU/mL after 24 weeks of Peg-IFNα therapy should stop using IFN (3). Even if they remained receiving IFN therapy, patients whose levels of HBsAg plateaued found it challenging to experience a continuous fall in the level of HBsAg, according to our earlier studies (9, 12, 20). Therefore, we did not create a control group for the Plateau group in this study.

The Ethics Committee of Beijing Ditan Hospital, which is a part of Capital Medical University, granted approval for this study (Jing Di Lun Ke Zi 2018 NO. 023-01), and it was registered as a clinical trial (NCT04028856). It was designed and implemented according to Helsinki standards. All selected patients have signed informed consent.

### Inclusion and exclusion criteria

Inclusion criteria (1): 18-65 years old; (2) HBsAg positive lasting more than 6 months (≥ 0.05 IU/mL). In addition, it was also necessary for Naïve group: (1) never received antiviral therapy; it was also necessary for NA-treated group: (1) so far and only take first-line oral antiviral therapy more than 6 months. For Plateau group, it also needed to be satisfied: (1) the level of HBsAg decreased to the plateau period after IFN antiviral treatment for more than 6 months. In order to be considered in a plateau period, the level of HBsAg must have dropped by 0.5 lg IU/mL from the preceding detection time point (more than 3 months) (12).

Exclusion criteria: (1) previously known viral hepatitis (except hepatitis B) and epstein-barr virus, cytomegalovirus, human immunodeficiency virus and other non-hepatitis virus infections; (2) complicated with other liver diseases, for instance metabolic-related fatty liver disease, alcoholic hepatitis, autoimmune hepatitis, hepatolenticular degeneration, decompensated cirrhosis and liver cancer; (3) mental illness; (4) severe heart, brain, lung, kidney or other system diseases; (5) usage of other liver-damaging drugs and/or chronic alcoholism; (6) usage of hormones and/or immunosuppressants.

### Peripheral blood mononuclear lymphocytes isolation

The peripheral venous blood of the patient was collected at the specified time point and put into the EDTA anticoagulation tube. Using lymphocyte separation solution, peripheral blood mononuclear lymphocytes (PBMC) were isolated by density gradient centrifugation. All samples were processed and analysed within 24 hours after sample collection.

### Flow cytometry analysis

PBMCs and direct coupling antibody were incubated together in dark environment at 4°C for 20 minutes. Then 2 mL of 1 × PBS were added to wash lymphocytes. After rotating 1200 rpm for 5 minutes, the supernatant was discarded. Finally, 200 mL of 1 × PBS was added to make the pre-test sample. The monoclonal antibodies were anti-human CD4-APC-Fire750 (Clone SK3), CD8-BV510 (Clone SK1), HLA-DR-PE-Cy7 (Clone L243), and CD38^-^APC (Clone HB-7) (BioLegend, San Diego, CA, USA). FACSCanto flow cytometry (BD Biosciences, San Diego, CA, USA) and FlowJo software (Tree Star, Ashland, OR, USA) was used to gather and analyze the data, respectively.

### Clinical indicators detection

Roche CobasTaqMan 96 (Roche, Pleasanton, CA, USA), an automatic real-time fluorescence quantitative polymerase chain reaction (PCR) detector, was utilized to detect serum HBV DNA load. Serum HBsAg and hepatitis B e antigen (HBeAg) levels were measured by Abbott Architect i2000 kits (Abbott Diagnostics, Abbott Park, IL, USA), and liver function indexes were detected by Hitachi 7600 automatic biochemical analyzer (Wako Pure Chemical Industries, Tokyo, Japan).

### Statistical analysis

SPSS 21.0 (IBM Corporation, Chicago, IL, USA) and GraphPad5 (GraphPad Software, La Jolla, CA, USA) software were used for statistical analysis. The categorical variables were expressed by frequency and percentage, and the chi-square test was used to compare the differences between groups. The normality of continuous variables was evaluated by Shapiro-Wilk test. Continuous variables that were normally distributed were represented by mean ± standard deviation (SD), and continuous variables that were not normally distributed were represented by the median and inter-quartile range (median, Q1-Q3). According to the normality of the baseline data, One-way ANOVA test and Kruskal-Wallis test were respectively used to compare the multiple groups at baseline, and then Tukey’s multiple comparisons test and Dunn’s multiple comparisons test were utilized to conduct pairwise comparisons between the groups. to make pairwise comparisons between the groups. The linear mixed effect model (restricted estimation maximum likelihood) was used to analyze the changes of parameters at different time points in each group, and the multiplicity of *P* value was adjusted by Bonferroni method. Patients were taken as random effects, groups and measurement time points were taken as fixed effects. The standard model was selected by likelihood ratio test. It did not violate the standard model hypothesis. The adjusted model took age and sex as covariables to exclude potential confounding factors. The Spearman correlation test was used to get the P values and correlation coefficient. All meaningful analyses were double-tailed, and *P* < 0.05 was statistically significant.

## Results

### Patient cohort

This study comprised 150 patients with CHB in total, including 53 patients in Naïve group, 51 patients in NA-treated group and 36 patients in Plateau group. **Table 1** displayed the initial demographic data of patients. Then, according to the wishes of the patients, 37 patients in the Naïve group were willing to start IFN treatment, collecting a total of 93 samples at 3 time points for follow-up detection and analysis, while 13 patients in the NA-treated group were willing to start using IFN on the basis of the original NA treatment, and a total of 33 samples were gathered at 3 different time points for follow-up analysis. 13 Plateau group patients were unable to complete the follow-up examination in our hospital, so a total of 56 samples were gathered from 23 patients at 3 time points for follow-up analysis.

**Table 1:** Comparison of baseline characteristics of the study population^a^.

Variables	Naïve group (n=53)	NA-treated group (n=51)	Plateau group (n=36)	*P*	*P* (Naïve *vs.* NA-treated)	*P* (Naïve *vs.* Plateau)	*P* (NA-treated *vs.* Plateau)
Age (years)	34.00 (31.50, 40.50)	42.00 (30.00, 50.00)	36.00 (32.25, 44.50)	0.0710	/	/	/
Man (%)	35 (66.04)	34 (66.67)	24 (66.67)	0.9971	/	/	/
Patient of HBeAg positive (%)	32 (60.38)	25 (49.02)	17 (47.22)	0.3747	/	/	/
HBsAg (log10 IU/mL)	3.65 ± 1.21	3.10 ± 0.86	2.34 ± 1.42	<0.0001	0.0680	<0.0001	0.0122
HBV DNA (log10 IU/mL)	4.94 ± 2.97	1.04 ± 1.28	0.84 ± 0.60	<0.0001	<0.0001	<0.0001	0.9019
ALT (U/L)	45.70 (31.40, 50.30)	22.00 (16.25, 25.50)	48.50 (45.00, 51.00)	<0.0001	0.0015	0.3170	<0.0001
AST (U/L)	31.30 (18.95, 50.90)	23.00 (19.00, 28.00)	28.95 (21.63, 39.58)	0.0060	0.0106	>0.9999	0.0395
HLA-DR^+^CD38^-^ CD4^+^ T lymphocytes (%)	4.16 (2.57, 6.35)	5.46 (3.82, 6.79)	3.04 (2.59, 3.37)	<0.0001	0.0289	0.0075	<0.0001
HLA-DR^+^CD38^dim^ CD4^+^ T lymphocytes (%)	2.79 (2.04, 4.15)	2.66 (1.91, 3.84)	5.90 (3.52, 9.49)	<0.0001	>0.9999	<0.0001	<0.0001
HLA-DR^+^CD38^hi^ CD4^+^ T lymphocytes (%)	0.38 (0.25, 0.62)	0.38 (0.26, 0.55)	3.11 (2.70, 3.67)	<0.0001	>0.9999	<0.0001	<0.0001
HLA-DR^+^CD38^-^ CD8^+^ T lymphocytes (%)	5.90 (2.64, 8.14)	11.70 (7.70, 15.20)	1.55 (0.93, 2.89)	<0.0001	0.0002	<0.0001	<0.0001
HLA-DR^+^CD38^dim^ CD8^+^ T lymphocytes (%)	9.05 (5.70, 13.35)	9.58 (5.93, 12.80)	5.02 (3.49, 7.41)	<0.0001	>0.9999	0.0003	<0.0001
HLA-DR^+^CD38^hi^ CD8^+^ T lymphocytes (%)	1.87 (0.95, 3.10)	1.51 (1.03, 2.37)	21.00 (16.07, 38.08)	<0.0001	>0.9999	<0.0001	<0.0001

NA, nucleoside (nucleotide) analogs; HBsAg, hepatitis B surface antigen; HBeAg, hepatitis B e antigen; HBV DNA, hepatitis B virus deoxyribonucleic acid; ALT, alanine aminotransferase; AST, aspartate aminotransferase; HLA-DR, human leukocyte antigen DR; SD, standard deviation; Q1, lower quartile; Q3, upper quartile.

a Values are expressed as No. (%) or mean ± SD or median (Q1, Q3).

### Baseline clinical characteristics of patients

In comparison to both the Naïve group and the NA-treated group, HBsAg amount in the Plateau group was much lower (*P* < 0.0001; *P* = 0.0122). Although the HBsAg level in the NA-treated group was marginally less than that in the Naïve group, there was no markedly significant variation (*P* = 0.0680). HBV DNA load was considerably higher in the Naïve group compared to the NA-treated group and Plateau group, respectively (*P* < 0.0001; *P* < 0.0001), but there was no remarkable variation in HBV DNA level between NA-treated and Plateau group (*P* = 0.9019). Alanine aminotransferase (ALT) and aspartate aminotransferase (AST) levels in NA-treated group were notably lower than those in Naïve group and Plateau group respectively (ALT: *P* = 0.0015; *P* < 0.0001; AST: *P* = 0.0106; *P* = 0.0395), but there was no discernible variation in terms of ALT and AST levels between Naïve group and Plateau group (ALT: *P* = 0.3170; AST: *P* > 0.9999), as shown in **Table 1**.

### Comparison HLA-DR and CD38 coexpression subsets on CD4^+^ and CD8^+^ T lymphocytes between groups at baseline

**Figure 1:**
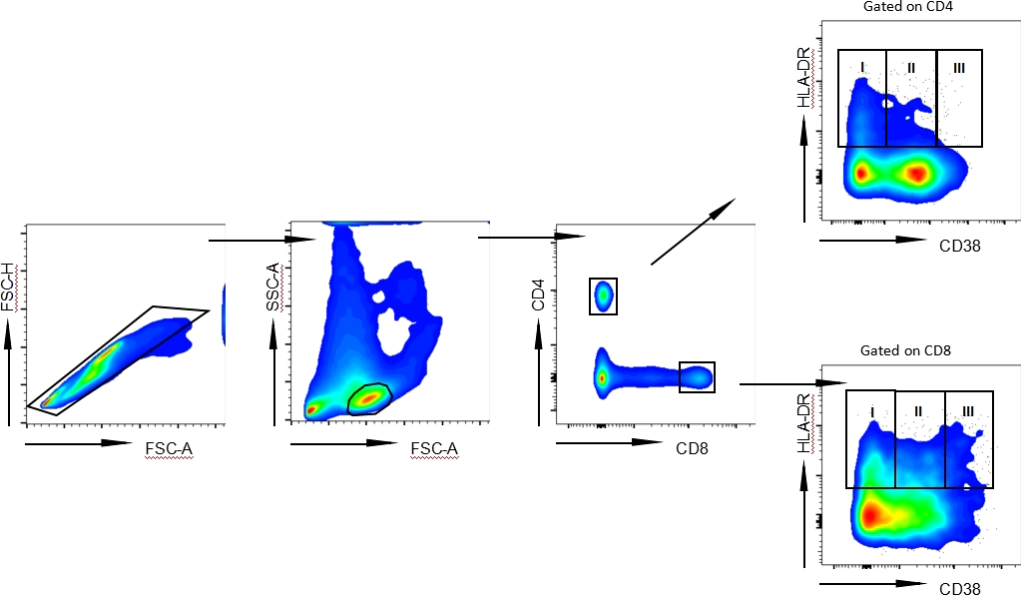
Representative flow cytometry gating strategy for HLA-DR^+^ subgroups on CD4^+^ and CD8^+^ T lymphocytes. Representative FACS pseudocolor showed three subpopulations of HLA-DR^+^ according to the expression of CD38 on CD4^+^ T lymphocytes and CD8^+^ T lymphocytes from CHB patient: HLA-DR^+^CD38^-^ (I), HLA-DR^+^CD38^dim^ (II) and HLA-DR^+^CD38^hi^ (III).

In order to determine the level of T lymphocyte activation under different treatment conditions, we analyzed the coexpression of HLA-DR and CD38, representing the key T lymphocyte activation indicator. We defined three subsets in activated HLA-DR^+^ CD4^+^ T and CD8^+^ T lymphocytes based on CD38 expression: HLA-DR^+^CD38^-^ subgroup, HLA-DR^+^CD38^dim^ subgroup and HLA-DR^+^CD38^hi^ subgroup (**Figure 1**). The percentage of HLA-DR^+^CD38^-^ CD4^+^ T lymphocytes in Plateau group was much less than that in Naïve group and NA-treated group (*P* = 0.0075; *P* < 0.0001). In the NA-treated group, more HLA-DR^+^CD38^-^CD4^+^ T lymphocytes were present than those in the Naïve group (*P* = 0.0289). HLA-DR^+^CD38^dim^ CD4^+^ T lymphocyte percentage in Plateau group was considerably greater than that in Naïve and NA-treated group (*P* < 0.0001; *P* < 0.0001), but no discernible variation was observed between Naïve and NA-treated group (*P* > 0.9999). The proportion of HLA-DR^+^CD38^hi^ CD4^+^ T lymphocytes in Plateau group was considerably greater than that in Naïve and NA-treated group (*P* < 0.0001; *P* < 0.0001), but there was no discernible variation in the percentage of HLA-DR^+^CD38^hi^ CD4^+^ T lymphocytes between Naïve group and NA-treated group (*P* > 0.9999). HLA-DR^+^CD38^dim^ CD8^+^ T lymphocyte percentage in Plateau group was noticeably less than that in Naïve group and NA-treated group, respectively (*P* = 0.0003; *P* < 0.0001), however there was also no discernible variation in HLA-DR^+^CD38^dim^ CD8^+^ T lymphocyte level between Naïve group and NA-treated group (*P* > 0.9999). The difference trend of HLA-DR^+^CD38^-^ CD8^+^ T lymphocytes and HLA-DR^+^CD38^hi^ CD8^+^ T lymphocytes in different groups was similar to that of CD4^+^ T lymphocytes. HLA-DR^+^CD38^-^ CD8^+^ T lymphocyte percentage in Plateau group was considerably lower than that in Naïve and NA-treated group (*P* < 0.0001; *P* < 0.0001). In NA-treated group, HLA-DR^+^CD38^-^ CD8^+^ T lymphocyte percentage was noticeably more than that in Naïve group (*P* = 0.0002). In comparison to the Plateau group, the HLA-DR^+^CD38^hi^ CD8^+^ T lymphocyte percentage was considerably less in Naïve group and NA-treated group (*P* < 0.0001; *P* < 0.0001). HLA-DR^+^CD38^hi^ CD8^+^ T lymphocyte proportion was not markedly different between Naïve group and NA-treated group (*P* > 0.9999), as shown in **Table 1**. Our data initially showed that the phenomenon that the antiviral efficacy of patients with long-term use of IFN could not continue was related to the change of HLA-DR and CD38 coexpression subsets percentage on CD4^+^ and CD8^+^ T lymphocytes after long-term IFN treatment.

### Comparison HLA-DR and CD38 coexpression subsets on CD4^+^ and CD8^+^ T lymphocytes during IFN treatment

In order to further explore the dynamic changes in HLA-DR and CD38 coexpression subsets on CD4^+^ and CD8^+^ T lymphocytes during IFN therapy, we followed up these patients who chose to start antiviral therapy with IFN in Naïve group and NA-treated group. Compared with those before IFN treatment, there was no noteworthy variation in the percentage of HLA-DR^+^CD38^-^ CD4^+^ T lymphocytes in Naïve group and NA-treated group after IFN treatment (Naïve group: 0 week *vs.* 4 week *P* > 0.9999; 0 week *vs.* 12-24 week *P* = 0.7210; NA-treated group: 0 week *vs.* 4 week *P* > 0.9999; 0 week *vs.* 12-24 week *P* > 0.9999). In both Naïve group and NA-treated group, HLA-DR^+^CD38^dim^ CD4^+^ T lymphocytes proportion increased considerably after 4 weeks of IFN treatment (*P* = 0.0020; *P* = 0.0010). HLA-DR^+^CD38^dim^ CD4^+^ T lymphocyte percentage in the Naïve group after 12-24 weeks of IFN treatment did not differ dramatically from that after 4 weeks of IFN treatment (*P* = 0.2020), but the HLA-DR^+^CD38^dim^ CD4^+^ T lymphocyte percentage in NA-treated group was dramatically less than that in IFN treatment for 4 weeks (*P* = 0.0090). The change trend in HLA-DR^+^CD38^hi^ CD4^+^ T lymphocyte percentage was consistent in Naïve group and NA-treated group during the treatment of IFN. At the beginning of treatment, HLA-DR^+^CD38^hi^ CD4^+^ T lymphocyte percentage in both groups increased compared with the baseline, but there was no discernible variation (*P* = 0.3500; *P* = 0.5110). In the late stage of IFN treatment, HLA-DR^+^CD38^hi^ CD4^+^ T lymphocyte percentage in both groups was dramatically higher than that at baseline (*P* < 0.0001; *P* < 0.0001). In Naïve group and NA-treated group, the proportion of HLA-DR^+^CD38^-^ CD8^+^ T lymphocytes decreased gradually with the increase of IFN treatment time (Naïve group: 0 week *vs.* 4 week *P* = 0.0170; 0 week *vs.* 12-24 week *P* < 0.0001; NA-treated group: 0 week *vs.* 4 week *P* = 0.0010; 0 week *vs.* 12-24 week *P* < 0.0001). In both Naïve group and NA-treated group, the proportion of HLA-DR^+^CD38^dim^ CD8^+^ T lymphocytes increased notably after 4 weeks of IFN treatment (*P* < 0.0001; *P* < 0.0001). After 12-24 weeks of IFN treatment, the percentage of HLA-DR^+^CD38^dim^ CD8^+^ T lymphocytes decreased notably compared with that of IFN treatment for 4 weeks in both groups (*P* < 0.0001; *P* < 0.0001). Moreover, with the increase of IFN treatment, the change trend of HLA-DR^+^CD38^hi^ CD8^+^ T lymphocyte percentage in Naïve group and NA-treated group was similar, which increased significantly with the duration of treatment (Naïve group: 0 week *vs.* 4 week *P* < 0.0001; 0 week *vs.* 12-24 week *P* < 0.0001; NA-treated group: 0 week *vs.* 4 week *P* < 0.0001; 0 week *vs.* 12-24 week *P* < 0.0001) (**Table 2**).

**Table 2:** The changes of indicators in each group during IFN treatment^a^.

Variables	Naïve group						NA-treated group					
	0 week (Baseline) (n=37)	4 week (IFN treatment) (n=34)	12-24 week (IFN treatment) (n=22)	*P* (0 week *vs.* 4 week)	*P* (0 week *vs.* 12-24 week)	*P* (4 week *vs.* 12-24 week)	0 week (Baseline) (n=13)	4 week (IFN treatment) (n=10)	12-24 week (IFN treatment) (n=10)	*P* (0 week *vs.* 4 week)	*P* (0 week *vs.* 12-24 week)	*P* (4 week *vs.* 12-24 week)
HBsAg (log10 IU/mL)	3.60 ± 1.29	/	2.79 ± 1.26	/	0.0010	/	3.48 ± 1.05	/	2.47 ± 1.06	/	0.0270	/
HBV DNA (log10 IU/mL)	5.19 ± 2.92	/	1.05 ± 0.66	/	<0.0001	/	1.98 ± 1.33	/	1.28 ± 0.94	/	0.3240	/
ALT (U/L)	48.86 ± 37.83	72.16 ± 45.35	47.53 ± 34.32	0.0250	>0.9999	0.0380	29.89 ± 14.27	72.39 ± 43.46	31.80 ± 8.28	0.0310	>0.9999	0.0420
AST (U/L)	33.19 ± 20.56	49.84 ± 26.34	43.05 ± 31.17	0.0080	0.8370	0.3090	24.04 ± 6.50	50.90 ± 17.35	34.00 ± 17.68	0.0420	>0.9999	0.4570
HLA-DR^+^CD38^-^ CD4^+^ T lymphocytes (%)	4.01 ± 2.19	4.02 ± 2.45	4.62 ± 2.14	>0.9999	0.7210	0.5740	4.52 ± 2.20	4.33 ± 2.38	4.07 ± 1.83	>0.9999	>0.9999	>0.9999
HLA-DR^+^CD38^dim^ CD4^+^ T lymphocytes (%)	3.42 ± 2.03	4.66 ± 2.03	5.50 ± 1.79	0.0020	<0.0001	0.2020	3.00 ± 1.41	5.34 ± 3.09	3.23 ± 0.79	0.0010	>0.9999	0.0090
HLA-DR^+^CD38^hi^ CD4^+^ T lymphocytes (%)	0.44 ± 0.27	0.79 ± 0.47	2.32 ± 2.03	0.3500	<0.0001	<0.0001	0.36 ± 0.23	0.87 ± 0.60	2.07 ± 0.12	0.5110	<0.0001	0.0110
HLA-DR^+^CD38^-^ CD8^+^ T lymphocytes (%)	5.59 ± 4.42	3.71 ± 2.50	2.22 ± 0.59	0.0170	<0.0001	0.0640	11.14 ± 7.12	6.10 ± 2.72	4.62 ± 2.98	0.0010	<0.0001	0.0160
HLA-DR^+^CD38^dim^ CD8^+^ T lymphocytes (%)	10.40 ± 7.94	14.89 ± 6.04	7.79 ± 3.35	<0.0001	0.0940	<0.0001	9.61 ± 4.57	17.62 ± 2.27	8.06 ± 3.12	<0.0001	>0.9999	<0.0001
HLA-DR^+^CD38^hi^ CD8^+^ T lymphocytes (%)	2.23 ± 1.95	7.69 ± 6.39	18.57 ± 9.74	<0.0001	<0.0001	<0.0001	2.08 ± 1.11	11.8 ± 2.98	20.02 ± 4.23	<0.0001	<0.0001	0.0020

NA, nucleoside (nucleotide) analogs; HBsAg, hepatitis B surface antigen; HBV DNA, hepatitis B virus deoxyribonucleic acid; ALT, alanine aminotransferase; AST, aspartate aminotransferase; HLA-DR, human leukocyte antigen DR; SD, standard deviation.

a Values are expressed as mean ± SD.

The aggregate data showed that there was no significant change in HLA-DR^+^CD38^-^ on CD4^+^ T lymphocytes in both groups during the treatment of IFN, but the proportion of CD8^+^ T lymphocytes decreased gradually. Except that the percentage of HLA-DR^+^CD38^dim^ CD4^+^ T lymphocytes in Naïve group did not change significantly at the later stage of IFN treatment, the changes of other HLA-DR^+^CD38^dim^ subsets on CD4^+^ and CD8^+^ T lymphocytes in both groups increased markedly first and then decreased gradually. Meanwhile, the proportion of HLA-DR^+^CD38^hi^ on both CD4^+^ and CD8^+^ T lymphocytes increased significantly as the increase of IFN treatment time.

### Comparison of clinical characteristics during IFN treatment

In Naïve group and NA-treated group, the HBsAg levels of all samples collected at 12-24 weeks after IFN treatment decreased more than 0.5 lg IU/mL compared with their respective treatment baselines. In Naïve group, compared with the baseline, HBsAg and HBV DNA levels declined considerably at 12-24 weeks after IFN treatment (*P* = 0.0010; *P* < 0.0001). At 12-24 weeks after IFN treatment, the level of HBsAg in NA-treated group declined markedly when compared to the baseline (*P* = 0.0270), but HBV DNA load had no significant change compared with the baseline (*P* = 0.3240). ALT and AST levels in Naïve group and NA-treated group were markedly higher than their respective baselines early in the course of IFN treatment (ALT: *P* = 0.0250; *P* = 0.0310; AST: *P* = 0.0080; *P* = 0.0420), but there was no discernible change compared with the baseline in the late stage of IFN therapy (ALT: *P* > 0.9999; *P* > 0.9999; AST: *P* = 0.8370; *P* > 0.9999), as shown in **Table 2**.

### Correlations between HBsAg and HLA-DR and CD38 coexpression subsets on CD4^+^ and CD8^+^ T lymphocytes during IFN treatment

**Figure 2:**
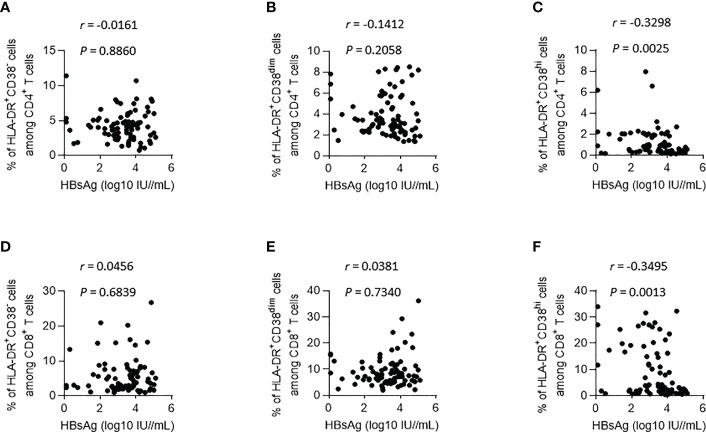
Correlations between HBsAg and HLA-DR^+^ subgroups (according to the expression of CD38) on CD4^+^ and CD8^+^ T lymphocytes during IFN Treatment. **(A–C)** Correlations between HBsAg and HLA-DR^+^CD38^-^ **(A)**, HLA-DR^+^CD38^dim^ **(B)** and HLA-DR^+^CD38^hi^ **(C)** on CD4^+^ T lymphocytes. **(D–F)** Correlations between HBsAg and HLA-DR^+^CD38^-^ **(D)**, HLA-DR^+^CD38^dim^ **(E)** and HLA-DR^+^CD38^hi^ **(F)** on CD8^+^ T lymphocytes.

Then, we used the longitudinal data collected from all patients in both groups to analyze the correlation between HBsAg and HLA-DR and CD38 coexpression subsets on CD4^+^ and CD8^+^ T lymphocytes (**Figure 2**). The proportion in HLA-DR^+^CD38^hi^ CD4^+^ T lymphocytes and HLA-DR^+^CD38^hi^ CD8^+^ T lymphocytes was adversely linked with HBsAg level, respectively (*R* = -0.3298, *P* = 0.0025; *R* = -0.3495, *P* = 0.0013). HLA-DR^+^CD38^-^ and HLA-DR^+^CD38^dim^ subset proportions on CD4^+^ and CD8^+^ T lymphocytes were not linked with HBsAg level. Collectively, these findings suggested that when the patients did not enter the plateau phase, the proportion of HLA-DR^+^CD38^hi^ CD4^+^ T lymphocytes and HLA-DR^+^CD38^hi^ CD8^+^ T lymphocytes increased gradually with the decrease of HBsAg level during IFN treatment.

### Changes in HLA-DR and CD38 coexpression subsets on CD4^+^ and CD8^+^ T lymphocytes during IFN intermittent treatment

In order to further explore whether the persistent high proportion of HLA-DR^+^CD38^hi^ subset on CD4^+^ and CD8^+^ T lymphocytes played a role in the plateau phase following long-term treatment of IFN, we continued to analyze the data of patients in the Plateau group after IFN intermittent treatment and IFN retreatment. Our data showed that after 12-24 weeks of IFN intermittent therapy, the proportion of HLA-DR^+^CD38^-^ CD4^+^ T lymphocytes (*P* < 0.0001) and HLA-DR^+^CD38^dim^ CD4^+^ T lymphocytes (*P* < 0.0001) increased significantly compared with the plateau baseline, while the HLA-DR^+^CD38^hi^ CD4^+^ T lymphocyte percentage decreased significantly compared with the plateau baseline (*P* < 0.0001). After 12-24 weeks of IFN intermittent treatment, the corresponding subsets of CD8^+^ T lymphocytes also had a similar pattern. Compared with the plateau baseline, the proportion of HLA-DR^+^CD38^-^ CD8^+^ T lymphocytes (*P* < 0.0001) and HLA-DR^+^CD38^dim^ CD8^+^ T lymphocytes (*P* < 0.0001) increased significantly, while the proportion of HLA-DR^+^CD38^hi^ CD8^+^ T lymphocytes decreased markedly (*P* < 0.0001) after IFN intermittent treatment with 12-24 weeks. In comparison to IFN intermittent treatment, the proportion of HLA-DR^+^CD38^-^ CD4^+^ T lymphocytes and HLA-DR^+^CD38^dim^ CD4^+^ T lymphocytes decreased markedly after 12-24 weeks of IFN retreatment (*P =* 0.0010; *P* < 0.0001). However, compared with plateau baseline, there were no noteworthy variation in both the proportion of HLA-DR^+^CD38^-^ CD4^+^ T lymphocytes and HLA-DR^+^CD38^dim^ CD4^+^ T lymphocytes after IFN retreatment (*P* > 0.9999; *P* = 0.6840). After 12-24 weeks of IFN retreatment, HLA-DR^+^CD38^hi^ CD4^+^ T lymphocyte percentage was considerably more than that after IFN intermittent treatment (*P =* 0.0130), but dramatically lower than that at the plateau baseline (*P* = 0.0180). Likewise, HLA-DR^+^CD38^-^ and HLA-DR^+^CD38^dim^ subsets proportion on CD8^+^ T lymphocytes after 12-24 weeks of IFN retreatment was greatly lower than that after IFN intermittent treatment, respectively (*P* < 0.0001; *P* < 0.0001), but there was no discernible change compared with the plateau baseline (*P* > 0.9999; *P* > 0.9999). Furthermore, HLA-DR^+^CD38^hi^ CD8^+^ T lymphocyte proportion increased considerably following IFN retreatment compared to IFN intermittent treatment. (*P =* 0.0060), but considerably lower than that at plateau baseline (*P* = 0.0160) (**Table 3**).

**Table 3:** The changes of indicators in Plateau group during IFN intermittent treatment and IFN retreatment^a^.

Variables	0 week (Baseline) (n=23)	12-24 week (IFN treatment interval) (n=15)	12-24 week (IFN retreatment) (n=18)	*P* (0 week *vs.* 12-24 week IFN treatment interval)	*P* (0 week *vs.* 12-24 week IFN retreatment)	*P* (12-24 week IFN treatment interval *vs.* 12-24 week IFN retreatment)
HBsAg (log10 IU/mL)	2.25 ± 1.46	2.32 ± 1.46	1.25 ± 1.29	>0.9999	0.0060	0.0030
HBV DNA (log10 IU/mL)	0.77 ± 0.65	0.52 ± 0.50	0.60 ± 0.57	0.6950	>0.9999	>0.9999
ALT (U/L)	50.39 ± 8.52	21.61 ± 6.95	52.00 ± 11.60	<0.0001	>0.9999	<0.0001
AST (U/L)	33.72 ± 12.56	15.68 ± 1.54	36.25 ± 4.22	<0.0001	>0.9999	<0.0001
HLA-DR^+^CD38^-^ CD4^+^ T lymphocytes (%)	5.18 ± 3.22	9.11 ± 6.90	6.48 ± 3.77	<0.0001	>0.9999	0.0010
HLA-DR^+^CD38^dim^ CD4^+^ T lymphocytes (%)	6.95 ± 4.45	9.35 ± 3.45	6.86 ± 3.83	<0.0001	0.6840	<0.0001
HLA-DR^+^CD38^hi^ CD4^+^ T lymphocytes (%)	3.35 ± 0.80	0.32 ± 0.25	2.00 ± 2.59	<0.0001	0.0180	0.0130
HLA-DR^+^CD38^-^ CD8^+^ T lymphocytes (%)	2.02 ± 2.02	6.46 ± 3.16	1.90 ± 1.36	<0.0001	>0.9999	<0.0001
HLA-DR^+^CD38^dim^ CD8^+^ T lymphocytes (%)	5.18 ± 2.77	26.93 ± 21.05	4.94 ± 1.85	<0.0001	>0.9999	<0.0001
HLA-DR^+^CD38^hi^ CD8^+^ T lymphocytes (%)	25.73 ± 15.58	3.13 ± 1.73	15.89 ± 7.53	<0.0001	0.0160	0.0060

HBsAg, hepatitis B surface antigen; HBV DNA, hepatitis B virus deoxyribonucleic acid; ALT, alanine aminotransferase; AST, aspartate aminotransferase; HLA-DR, human leukocyte antigen DR; SD, standard deviation.

a Values are expressed as mean ± SD.

### Changes of clinical characteristics during IFN intermittent treatment

There was no significant variation in HBsAg level in Plateau group before and after IFN intermittent treatment (*P* > 0.9999). HBsAg level of each sample collected after IFN retreatment in Plateau group declined more than 0.5 lg IU/mL compared with that after IFN intermittent treatment, which was statistically noteworthy (*P* = 0.0030), and considerably lower than that at plateau baseline as well (*P* = 0.0060). During the IFN intermittent treatment and IFN retreatment, HBV DNA load in Plateau group had no significant change (0 week *vs.* 12-24 week IFN treatment interval *P* = 0.6950; 12-24 week IFN treatment interval *vs.* 12-24 week IFN retreatment *P* > 0.9999). In comparison to the baseline of plateau phase, ALT (*P* < 0.0001) and AST (*P* < 0.0001) levels in Plateau group decreased significantly after IFN intermittent treatment, while compared with those after IFN intermittent treatment, ALT (*P* < 0.0001) and AST levels(*P* < 0.0001) increased considerably in Plateau group after IFN retreatment, as shown in **Table 3**.

## Discussion

The coexpression of CD38 and HLA-DR was associated with T lymphocyte activation. Many studies have shown HLA-DR^+^CD38^+^ T lymphocytes played an effective role in immune activation and virus clearance (21, 22). However, some studies (23, 24) have illustrated that activated HLA-DR^+^CD38^+^ T lymphocytes were closely related to the severity of the disease. Du et al. (25) further explored the HLA-DR^+^CD38^+^ on T lymphocytes in patients with acute coronavirus disease 2019 infection, and it was found that there were two heterogeneous subsets of HLA-DR^+^CD38^+^ on T lymphocytes: HLA-DR^+^CD38^dim^ and HLA-DR^+^CD38^hi^. HLA-DR^+^CD38^dim^ subsets expressed low level of inhibition checkpoints and strong cytotoxic potential, and were less sensitive to apoptosis. HLA-DR^+^CD38^hi^ subsets have been proved to be in a state of immune disorder of overactivation or depletion, and the cytotoxic function was impaired. Although patients with CHB had a chronic virus infection, IFN could activate immune lymphocytes, enhancing their secretion of cytokines and killing function (26). Therefore, we also focused on how HLA-DR and CD38 coexpression subsets changed dynamically on T lymphocytes following IFN-based CHB therapy.

CD4^+^ T lymphocytes helped activate B lymphocytes to produce neutralizing antibodies and induced cytotoxic T lymphocytes (CTL) responses, while CD8^+^ T lymphocytes contributed to the generation of antiviral cytokines, such as IFN-γ and tumor necrosis factor-α, and could differentiate into CTL lymphocytes to exert cytotoxic activity to clear virus-infected hepatocytes (27). Through the comparison of the proportion of CD4^+^ T and CD8^+^ T subsets in three groups of patients under different treatment background at the baseline, it could be found that for both CD4^+^ T and CD8^+^ T lymphocytes, long-term IFN treatment significantly decreased the proportion of HLA-DR^+^CD38^-^ subsets and dramatically increased the proportion of HLA-DR^+^CD38^hi^ subsets. However, the results of HLA-DR^+^CD38^dim^ subsets in CD4^+^ and CD8^+^ T lymphocytes were slightly different. At this time, the dynamic changes of subsets in Naïve and NA-treated group during IFN treatment were analyzed, and it was found that the proportion of HLA-DR^+^CD38^-^ CD8^+^ T lymphocytes decreased gradually during IFN treatment, but the change in HLA-DR^+^CD38^-^ CD4^+^ T lymphocyte percentage during treatment was not significant. Actually, this abnormal phenomenon might be associated with the following reasons: it could be seen from the data of this study that the percentage change of each subgroup in CD4^+^ T lymphocytes was slighter than that in CD8^+^ T lymphocytes during IFN treatment. In the course of IFN treatment, HLA-DR^+^CD38^-^ CD4^+^ T lymphocyte proportion in NA-treated group decreased gradually, although there was no statistical difference. In addition, it might also be related to the large dispersion of samples. In the course of IFN treatment, the change trend of HLA-DR^+^CD38^dim^ subsets which played a positive function of virus clearance was found to be significantly increased at first, and then gradually decreased. This trend was most typical in CD8^+^ T lymphocytes, which was consistent with earlier stydies (26, 28). During IFN treatment in Naïve group, and the comparison between three groups at first at the baseline, the abnormal phenomenon of HLA-DR^+^CD38^dim^ CD4^+^ T lymphocyte percentage could be explained as the long-term treatment of IFN eventually led to the decrease of HLA-DR^+^CD38^dim^ subgroup percentage on T lymphocytes, which was based on the increase of the percentage of this subgroup in the early stage of IFN treatment. At this time, reduced HLA-DR^+^CD38^dim^ CD4^+^ T lymphocyte subsets percentage was still higher than that at baseline. The proportion of overactivated and exhausted HLA-DR^+^CD38^hi^ subsets increased significantly during IFN treatment for both CD4^+^ and CD8^+^ T lymphocytes.

Moreover, HBsAg level in Naïve group and NA-treated group decreased significantly during IFN treatment. ALT and AST levels increased significantly in the initial stage of IFN treatment, which was linked with the enhancement of T lymphocyte immunological performance and NK lymphocyte activity after IFN treatment, indicating a better antiviral effect (29). The correlation between virological index and the proportion of HLA-DR and CD38 coexpression subsets on T lymphocytes was analyzed. The results revealed that when the patients did not reach the plateau stage of IFN, the level of HBsAg was significantly linked with the proportion of HLA-DR^+^CD38^hi^ subsets. With the extension of IFN treatment time, the proportion of HLA-DR^+^CD38^hi^ subsets of both CD4^+^ and CD8^+^ T lymphocytes would gradually increase as the HBsAg level fell. In summary, our research showed that in the process of IFN treatment, the percentage of HLA-DR^+^CD38^dim^ subsets that played a positive virus clearance function was significantly increased in the initial stage of IFN treatment, which was helpful to quickly eliminate the virus and reduce the level of HBsAg. At the same time, HLA-DR^+^CD38^dim^ subsets gradually differentiated into HLA-DR^+^CD38^hi^ subsets, which represented depletion. In the late course of IFN treatment, HLA-DR^+^CD38^dim^ subset percentage decreased, while the percentage of HLA-DR^+^CD38^hi^ subsets increased gradually.

The plateau phase occurred after long-term treatment of IFN, which meant that the antiviral effect of IFN could not be continued. Some studies have shown that it might be related to the gradual decrease of IFN receptor expression (30), the production of endogenous IFN antibodies (31) which decreased or eliminated IFN efficacy or affected the normal clearance and degradation of IFN, and the significant decrease of the absolute number of CD8^+^ T lymphocytes (19). Whether the occurrence of the IFN plateau phase and the effectiveness of intermittent therapy can also be explained by the dynamic changes of HLA-DR and CD38 coexpression subsets during the long-term treatment of IFN is worthy of further discussion. Surprisingly, compared with the baseline of IFN plateau, after pausing the use of IFN for 12-24 weeks, the subsets of HLA-DR^+^CD38^-^ and HLA-DR^+^CD38^dim^ on CD4^+^ and CD8^+^ T lymphocytes elevated significantly, while HLA-DR^+^CD38^hi^ subsets decreased significantly. After IFN retreatment with 12-24 weeks, HLA-DR^+^CD38^-^ and HLA-DR^+^CD38^dim^ subsets decreased significantly, while HLA-DR^+^CD38^hi^ subsets increased significantly, which was consistent with the above demonstration. Before and after retreatment of IFN, the level of HBsAg declined significantly, while the level of ALT and AST elevated significantly, which also meant that IFN began to exert its antiviral effect and further enhance the function of immune lymphocytes (6, 29). Meanwhile, even if IFN began to restore antiviral efficacy, there was no noticeable variation in the percentage of HLA-DR^+^CD38^-^ and HLA-DR^+^CD38^dim^ subsets on CD4^+^ and CD8^+^ T lymphocytes after IFN retreatment compared with the baseline of IFN plateau phase. At this time, the proportion of HLA-DR^+^CD38^hi^ subsets on CD4^+^ and CD8^+^ T lymphocytes was dramatically lower than that at the baseline of plateau phase, respectively. In a word, the persistent high proportion of HLA-DR^+^CD38^hi^ subsets on CD4^+^ and CD8^+^ T lymphocytes was related to the occurrence of IFN plateau phase. After IFN intermittent treatment, the proportion of HLA-DR^+^CD38^hi^ subsets decreased, suggesting that the immune disorder of T lymphocyte overactivation may be restored. During the IFN retreatment, the proportion of HLA-DR^+^CD38^hi^ subsets increased gradually, but it did not reach the higher level compared with the baseline of plateau phase. Therefore, IFN could continue to play an antiviral role and HBsAg began to decline again.

Our study had several drawbacks, including a small sample size and lack of enrollment of patients who participated in the whole period from initial IFN treatment to the IFN plateau stage. More importantly, the exact functional properties of HLA-DR^+^CD38^dim^ and HLA-DR^+^CD38^hi^ subsets on T lymphocytes could not be determined without further functional verification. Therefore, more evidence is needed to verify the role of HLA-DR and CD38 coexpression subsets on T lymphocytes when treating CHB using IFN.

## Conclusion

In summary, our study demonstrated that the HLA-DR and CD38 coexpression subsets on T lymphocytes dynamically changed over the course of receiving long-term IFN treatment. The appearance of IFN plateau phase was related to the persistent high proportion of HLA-DR^+^CD38^hi^ subsets on T lymphocytes. IFN intermittent treatment could significantly reduce the proportion of HLA-DR^+^CD38^hi^ subsets, helping regain the antiviral efficacy of IFN during IFN retreatment.

## Data availability statement

The raw data supporting the conclusions of this article will be made available by the authors, without undue reservation.

## Ethics statement

The studies involving human participants were reviewed and approved by the Ethics Committee of Beijing Ditan Hospital Affiliated with Capital University of Medical Sciences. The patients/participants provided their written informed consent to participate in this study.

## Author contributions

RH, ML and YX contributed to study concept and design. YLi, XB and LY conducted experiments and collected the data. YLi, GS, SX, HL, XB and LY collected the information of the patients. TJ, WD, SWa, LZ, YLu and YG ordered laboratory materials. YLi performed the statistical results and wrote the first draft. HH, SWu, RL, MC, MX, LH and XC edited the English version. All authors contributed to the article and approved the submitted version.
